# Medical student acceptance on gene therapy to increase children's well-being with genetic diseases: a study in Indonesia

**DOI:** 10.2144/fsoa-2021-0130

**Published:** 2022-05-30

**Authors:** Dimas Setyanto, Annette d'Arqom, Danti Nur Indiastuti, Ema Qurnianingsih, Nurina Hasanatuludhhiyah, Safira Nur Izzah, Mhd Zamal Nasution, Junaidah Yusof

**Affiliations:** 1 Medical Program, Faculty of Medicine, Universitas Airlangga, Surabaya, 60131, Indonesia; 2 Department of Anatomy, Histology, & Pharmacology, Faculty of Medicine, Universitas Airlangga, Surabaya, 60131, Indonesia; 3 Translational Medicine & Therapeutic Research Group, Universitas Airlangga, Surabaya, 60131, Indonesia; 4 Department of Physiology & Medical Biochemistry, Faculty of Medicine, Universitas Airlangga, Surabaya, 60131, Indonesia; 5 Postgraduate School, Universitas Airlangga, Surabaya, 60286, Indonesia; 6 Institute for Population & Social Research, Mahidol University, Bangkok, 73170, Thailand; 7 School of Human Resource Development & Psychology, Faculty of Social Sciences & Humanities, Universiti Teknologi Malaysia, Johor Bahru, 81310, Malaysia

**Keywords:** attitude, clinical, pre-clinical, quality of life, well-being

## Abstract

**Aim:**

Gene therapy is expected to improve patients' quality of life. Medical students need to be aware about this technology as its application is becoming wider.

**Materials & methods:**

A web-based survey was conducted to measure the acceptance of Indonesian medical students regarding gene therapy.

**Results:**

Data from 621 valid responses showed that Indonesian medical students have little knowledge of this technology, with 34.4% of them ever heard of gene therapy. However, most of them support the approved gene therapy for health-related matters, but not on the non-health related matters. Their acceptance was determined by the sex, domicile and studentship status.

**Conclusion:**

Increasing medical students' knowledge of gene therapy is important to minimize the future conflict of gene therapy application.

## Plain language summary

Gene therapy is a new breakthrough for the medical world that aims to improve children with genetic diseases, their family well-being and quality of life. There is a possibility that gene therapy will be used in Indonesia, therefore preparedness for adopting this new treatment is important for the future medical doctor. This study shows a low level of knowledge of Indonesian medical students on this technology, even though their acceptance for gene therapy application in health-related matters is high. Increasing medical students' knowledge of gene therapy is important to minimize the future conflict of gene therapy application.

## Graphical abstract

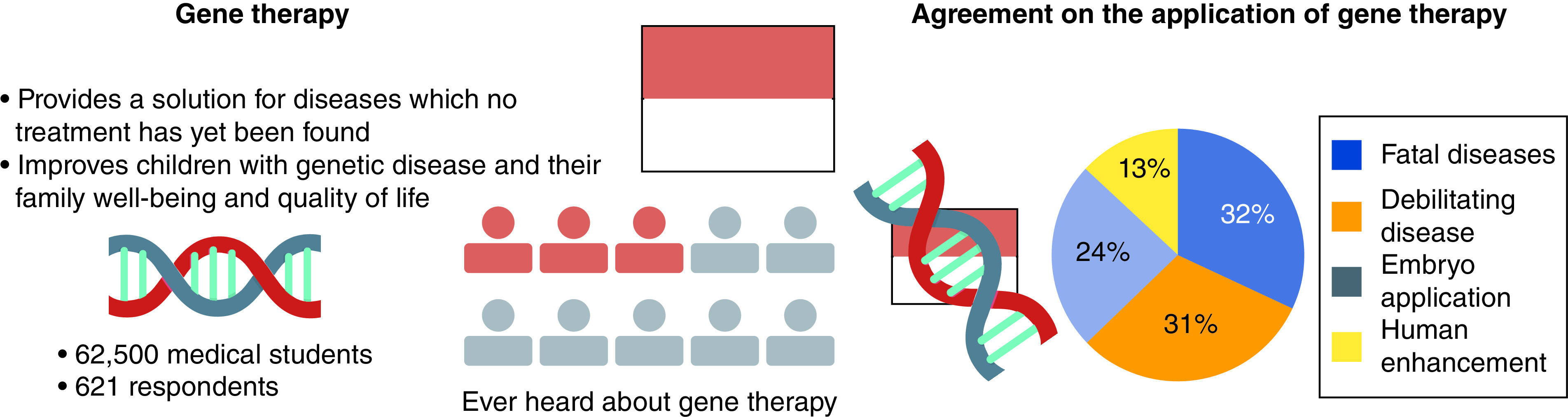

Genetic diseases cause physical, psychosocial and economic burdens for patients, family members and society. Reducing the quality of life and well-being of those affected has been reported in various genetic diseases, such as thalassemia [1–3], Duchenne muscular dystrophy (DMD) [4,5], retinal diseases [6], spinal muscular atrophy (SMA) [7,8], idiopathic pulmonary fibrosis [9], cystic fibrosis [10,11] and various cancers [12,13]. Most current therapies focus on reducing the symptoms of the diseases. However, finding curative treatments might bring benefits, including reducing the burden and increasing the quality of life.

Because mutations or gene deletion are the cause of genetic diseases, the option for curative treatment is to introduce the correct DNA/RNA into the cells, removing or changing defective genes to drive the correct protein production, which can be achieved using gene therapy. Gene therapy is an emerging experimental treatment that delivers functional genes in the human body to counter or replace malfunctioning genes; thus, curing diseases without pharmacologic intervention, radiotherapy or surgery [14]. This therapeutic strategy may be used to provide a functional gene among patients with a mutated non-functioning gene or an under-expressed gene. This technique can also be used to express proteins such as growth factors in enhancing cell survival [15]. Some gene therapies have been approved by the US FDA including Kymriah^®^ for leukemia [16], Luxturna^™^ for inherited retinal disease [17], Zolgensma^®^ for SMA [18], eteplirsen for DMD [19], Trikafta^®^ for cystic fibrosis [20] and Zynteglo^™^ for thalassemia which remain under consideration [21].

However, even though gene therapies provide a breakthrough solution for genetic diseases and potentially increase the quality of life and well-being of patients and family members, the cost and the access to the treatments become a major challenge [15,22]. Ethical issues regarding the safety and the risk of gene therapy also have become a concern [23,24]. Despite the controversies, medical students, as future users of this treatment, need to understand this technology to approve and develop this technology.

In Indonesia, a study among 1,054 medical students and doctors showed only 16% of the respondents knew about genome editing, which is one method of gene therapy involving genome modification. Moreover, the respondents were most likely to accept the application of genome editing for health purposes. Their acceptance is affected by various demographic factors [25]. Because the currently approved gene therapies do not involve modifying the genome, it would be interesting to understand the medical students' attitudes, as future users, regarding these approved gene therapies. Thus, we performed a cross-sectional web-based survey to investigate the attitude of medical students in Indonesia on the approved gene therapies which may potentially increase the quality of life and well-being of children with genetic diseases. Furthermore, the positive and negative predictors of their attitudes were analyzed.

## Material & methods

### Study design & data collection

This research was part of a study on gene therapy and genome editing in Indonesia. The web-based survey was conducted from May to December 2020 using a web-based survey (www.surveyplanet.com) which was distributed using email and social media, mainly WhatsApp and Facebook, to Indonesian medical students. A nonprobability sampling method, convenience sampling, was used in this study. The purpose of the study and neutral explanation about gene therapies were given on the landing page. Informed consent was obtained when the respondents clicked the BEGIN button of the survey. This study followed the Checklist for Reporting Results of Internet E-Surveys (CHERRIES) guidelines, including the prevention of multiple submissions from similar devices.

The inclusion criteria were Indonesian citizenship over 18 years old and enrolled in a medical program in an Indonesian university. The required minimum sample size was calculated using a sample size calculator (http://www.raosoft.com/samplesize.html) with a 5% margin of error, 95% confidence level, and an estimated number of medical students in Indonesia was 65,000. Thus, the minimum sample size required in this study was 382 respondents.

### Survey instrument

This instrument consisted of two parts: the respondents' basic information including sex, age, domicile, length of stay, religion, education, marital status, childbirth, economic status and study experience abroad; and closed-ended questions about their basic knowledge about gene therapies and attitudes toward gene therapy in embryos, adult cells, its application in certain conditions and its implementation in Indonesia. This questionnaire was adapted from Wang *et al.* [26] which was later translated to Bahasa Indonesian by two native Indonesian medical doctors and one Indonesian social science expert. The translated questionnaire was then pilot tested on 20 Indonesian medical students to ensure the validity of the questionnaire. The wording and sentences were evaluated based on the respondents' answer before collecting broader data.

### Analytical procedure

The data from the survey generator were extracted, verified and coded using Microsoft Excel, followed by analysis using SPSS 25.00 and visualized using GraphPad PRISM, Version 5.00 (CA, USA) and canva (www.canva.com). The respondents were categorized based on their sociodemographic characteristics with dichotomous variables. Domicile was categorized into two categories, i.e., resided in the most developed islands in Indonesia (Java and Bali) and in the less developed islands. Religion was categorized in the majority and non-majority, while self-assessed economic status was divided into average and above average.

Respondents' knowledge levels toward genetically modified food and approved gene therapies were measured using yes or no options. Moreover, respondents' attitudes toward gene therapy application on health and non-health related matters, and its application in Indonesia were measured using a 5-point Likert scale, assessed from strongly disagree, disagree, neutral, agree and strongly agree. An open-ended question on the factors influencing their attitudes toward gene therapies was asked at the end of the survey.

Descriptive statistical analysis was performed with the response rate calculated as a percentage on each item associated with the categorical variable. Inferential statistics was also performed with differences between groups measured using the chi-square test. To investigate the predictors of agreements to gene therapy, ordinal logistic regression with 95% confidence intervals (95% CIs) were calculated. All models were mutually adjusted for all potential confounders including sex, domicile, religion, clinical experience, economic status, student abroad experience, knowing someone with genetic diseases, and someone with diseases that limited daily life activities. Significance values were defined as p-value < 0.05.

## Results

### Characteristics of respondents

From the 647 responses received, 621 valid questionnaires were used in the final analysis, corresponding to an effective rate of 95.98%. Six responses were excluded due to their citizenship or student status, and 20 respondents were excluded due to unmet inclusion criteria. Respondents were divided into two groups based on their clinical experience, which were pre-clinical and clinical stage in medical school. Most respondents in both groups were female, with a female to male ratio of 2:1. All respondents were from 18 to 30 years old. Based on their domicile, as expected, 50.1% of pre-clinical students and 60.9% of clinical students lived in the more developed provinces where more medical schools were located compared with less developed provinces (49.9% and 39.1%; p < 0.029). Only 13.7% of respondents, with a third of the clinical students and less than one-tenth of pre-clinical students, had experience studying abroad which might have exposed them to more knowledge on gene therapy (p < 0.000). As expected, the clinical-stage students knew more people with fatal genetic diseases such as Down's syndrome, SMA, and thalassemia (60.9 vs 47.5%; p < 0.007); and people with a genetic disease that limited daily life activities such as Alzheimer's, dementia, and Parkinson's (52.3% vs 36.5%; p < 0.001) compared with pre-clinical medical students. The sociodemographic characteristics are summarized in Table 1.

**Table 1.** Sociodemographic characteristics of respondents.

Characteristic	Pre-clinical (n = 493)	Clinical (n = 128)	X^2^	p-value
Sex			1.215	0.270
Male	141 (28.6)	43 (33.6)		
Female	352 (71.4)	85 (66.4)		
Domicile			4.783	**0.029^†^**
Java and Bali Islands (more developed islands)	247 (50.1)	78 (60.9)		
Other Islands	246 (49.9)	50 (39.1)		
Religion			0.717	0.397
Majority	376 (76.3)	93 (72.7)		
Minority	117 (23.7)	35 (27.3)		
Economic status			0.403	0.525
Below and average	392 (79.5)	105 (82)		
Above	101 (20.5)	23 (18)		
Experience abroad			42.094	**0.000^§^**
No	448 (90.9)	88 (68.8)		
Yes	45 (9.1)	40 (31.2)		
Knowing patients with fatal disease			7.378	**0.007^‡^**
No	259 (52.5)	50 (39.1)		
Yes	234 947.5)	78 (60.9)		
Knowing patients with debilitating disease			10.634	**0.001^‡^**
No	313 (63.5)	61 (47.7)		
Yes	180 (36.5)	67 (52.3)		

† Statistically significant difference with a p-value < 0.05.

‡ Statistically significant difference with a p-value < 0.01.

§ Statistically significant difference with a p-value < 0.001.

### Familiarity with GT

**Figure 1.**
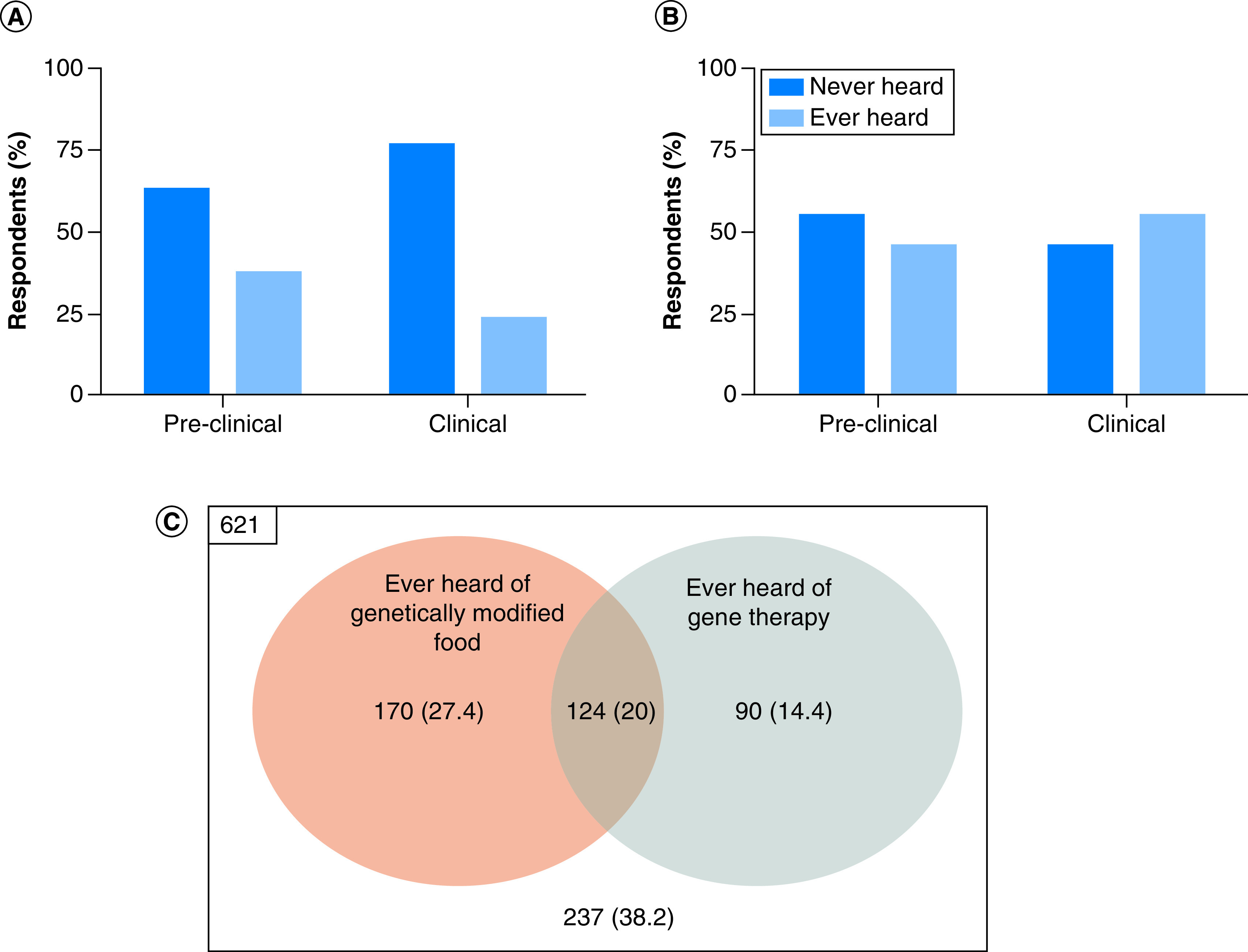
Respondents' familiarity with genetically modified food and approved gene therapy. Comparison of knowledge between pre-clinical and clinical students on **(A)** Gene therapy, **(B)** Genetically modified food. **(C)** Venn diagram of students' knowledge on gene therapy and genetic modified food.

Indonesian medical students were less familiar with the approved gene therapies, as only 37.3% of pre-clinical students and 23.4% clinical students (34.4% out of the total respondents) ever heard about this technology, such as Kymriah^®^ for leukemia treatment, Zynteglo^™^ for thalassemia, eteplirsen for DMD and nusinersen/Spiranza^®^ for SMA (Figure 1A). The respondents in both groups were more familiar with genetically modified food (45.4 and 54.7%, respectively; Figure 1B). From this number, only 20% of respondents ever heard of these genetic modified technologies, while 38.2% of them never heard about these issues (Figure 1C). These findings indicated that respondents were more aware of genetically modified food compared with gene therapy, even though the FDA has approved several gene therapies in recent years. Moreover, during the current pandemic, gene therapy was being discussed due to public concern regarding the effect of RNA and viral vector vaccines to alter the genome. Albeit most of the studies showed that those vaccines have no significant ability for genome integration, emerging studies showed other possibilities which need further investigation [27–30].

### Attitudes toward approved GT on genetic diseases & human enhancement

**Figure 2.**
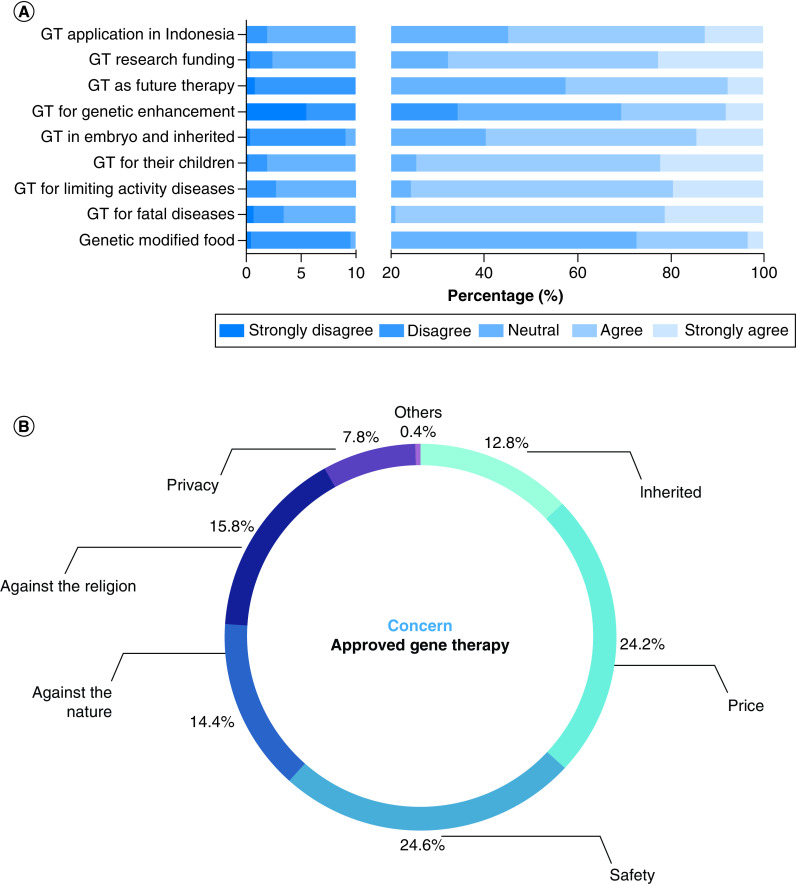
Acceptance and concern of Indonesian medical students on the approved gene therapy. **(A)** Acceptance on the approved gene therapy, **(B)** Medical students' concerns on approved gene therapy.

Further, the respondents were asked about their attitudes toward the approved gene therapies for fatal genetic diseases and debilitating genetic diseases. Short neutral explanations on each purpose or condition of the gene therapy were provided to each question. Thus, the respondents with less knowledge of this technology were still able to show their agreement. The answers were counted as 1 for strongly disagree, 2 for disagree, 3 for neutral, 4 for agree and 5 for strongly agree. Pre-clinical and clinical medical students' attitudes concerning gene therapy applications were compared using the Mann–Whitney U test (Table 2). Most respondents were neutral regarding genetically modified food technology, with only 27.33% agreeing in the pre-clinical and clinical students' groups (3.19 ± 0.66, 3.29 ± 0.69, respectively). However, the respondents in both groups reported more agreement toward gene therapy for health-related purposes, such as treating fatal diseases (79.07%; 3.95 ± 0.76 vs 4 ± 0.69) and genetic diseases caused limited activity (75.68%; 3.93 ± 0.72 vs 3.90 ± 0.7). The respondents in both groups (74.55%) also supported the application of approved gene therapies if they had children with genetic diseases (3.92 ± 0.74 vs 4.04 ± 0.73). Even though approved gene therapies could not be used in an embryo and for genetic enhancement purposes, their agreement on the possible future application of gene therapy was slightly reduced for embryo application (3.66 ± 0.83 vs 3.61 ± 0.89) with only 59.74% agreeing on this aim. Their approval also reduced drastically for genetic enhancement purposes with only 30.59% agreeing, especially in the clinical students' group (3.04 ± 1 vs 2.8 ± 1.1; p < 0.012). Overall, Indonesian medical students' attitudes on approved gene therapies are summarized in Table 2 and Figure 2A.

**Table 2.** Respondents' attitudes on the approved gene therapy technology.

Question	Pre-clinical (n = 493)	Clinical (n = 128)	Z	p-value
Q3. GMO	3.19 + 0.66	3.29 + 0.69	-1.486	0.137
Q4. GT for fatal diseases	3.95 + 0.76	4 + 0.69	-0.531	0.595
Q5. GT for debilitating diseases	3.93 ± 0.72	3.90 ± 0.7	-0.320	0.749
Q6. GT for children with genetic diseases	3.92 ± 0.74	4.04 ± 0.73	-1.694	0.090
Q7. GT in the embryo stage	3.66 ± 0.83	3.61 ± 0.89	-0.292	0.770
Q8. GT for human enhancement	3.04 ± 1	2.8 ± 1.1	-2.508	**0.012**
Q9. GT application in the future	3.38 ± 0.79	3.39 ± 0.85	-0.248	0.804
Q10. Funding GT research	3.87 ± 0.79	3.89 ± 0.76	-0.368	0.713
Q11. GT Application in Indonesia	3.63 ± 0.72	3.74 ± 0.71	-1.640	0.101

Bold text indicates a statistically significant difference with a p-value < 0.05.

GMO: Genetically modified organism; GT: Gene therapy.

### Support & concern for gene therapy

Respondents believed that gene therapy would be a ubiquitous technology in the future (3.38 ± 0.79 vs 3.39 ± 0.85), which might improve children's quality of life and well-being with genetic diseases. They also supported its application in Indonesia (3.63 ± 0.72 vs 3.74 ± 0.71) as well as funding to develop this technology (3.87 ± 0.79 vs 3.89 ± 0.76). Furthermore, respondents were asked their main concerns regarding this technology. The results indicated that safety was the greatest respondents' concern (24.6%), followed by the price (24.2%) and against religious values (15.8%). The respondents did not emphasize the privacy breach due to the application of this technology (Figure 2B).

### Determinants of support for gene therapy

To determine which factors corelated with their attitudes toward approved gene therapy, ordinal logistic regression analysis was performed. Results from modelling the ordinal outcome as a function of sex, domicile, student status, economic status, study abroad experience, knowing patients with fatal genetic diseases, knowing patients with limited daily life activity genetic diseases and their attitudes on genetically modified food are shown in Tables 3 & 4. The results showed that males were more likely to accept approved gene therapy for fatal diseases (OR: 2.07; CI: 1.469–2.927) and limited daily activity genetic diseases (OR: 2.15; CI: 1.523–3.025). Males also were a positive predictor to support gene therapy applications for their children (OR: 1.84; CI: 1.317–2.569) and embryo applications which could be inherited to the next generation (OR: 1.63; CI: 1.179–2.259). Moreover, respondents who did not know patients with limited daily activities were less likely to support the application to life-threatening genetic diseases (OR: 1.51; CI: 1.078–2.124). Students residing in the main or more developed islands were less likely (OR: 0.65; CI: 0.481–0.883) to approve gene therapy for genetic enhancement such as the appearance, intelligence, and strength, differing from pre-clinical medical students who were more likely to approve of gene therapy for this purpose (OR: 1.69; CI: 1.164–2.458). Male respondents (OR: 1.58; CI: 1.139–2.191) were more likely to have the positive point of view that gene therapy will become a future therapy, *vice versa* with respondents who had not experienced study abroad (OR: 0.38; CI: 0.238–0.616). Furthermore, students from developed islands were more likely to support funding for gene therapy development in Indonesia (OR: 1.51; CI: 1.103–2.062).

**Table 3.** Ordinal logistic regression analysis from attitudes of Indonesian medical students (Q3–Q8).

Parameters		Q3 genetic modified food			Q4 GT fatal diseases			Q5 GT limited daily activity			Q6 GT for children with genetic diseases			Q7 GT in embryo and inherited			Q8 GT for genetic enhancement		
		p-value	95% CI	OR	p-value	95% CI	OR	p-value	95% CI	OR	p-value	95% CI	OR	p-value	95% CI	OR	p-value	95% CI	OR
Gender	Males	**0.000** ^§^	(1.863, 1.314)	1.86	**0.000** ^§^	(1.469, 2.927)	2.07	**0.000** ^§^	(1.523, 3.025)	2.15	**0.000** ^§^	(1.317, 2.569)	1.84	**0.003** ^‡^	(1.179, 2.259)	1.63	0.369	(0.844, 1.579)	1.15
	Females	–	–		–	–		–	–		–	–		–	–		–	–	
Domicile	Main Islands	0.885	(0.975, 0.694)	0.98	0.679	(0.674, 1.293)	0.93	0.668	(0.674, 1.287)	0.93	0.816	(0.701, 1.324)	0.96	0.710	(0.691, 1.286)	0.94	**0.006** ^‡^	(0.481, 0.883)	0.65
	Other islands	–	–		–	–		–	–		–	–		–	–		–	–	
Faith	Majority	0.083	(0.490, 1.045)	0.72	0.108	(0.510, 1.068)	0.74	0.062	(0.488, 1.017)	0.71	0.698	(0.650, 1.333)	0.93	0.726	(0.662, 1.344)	0.94	0.341	(0.839, 1.660)	1.18
	Minority	–	–		–	–		–	–		–	–		–	–		–	–	
Studentship status	Pre-clinical	0.460	(0.567, 1.293)	0.86	0.842	(0.644, 1.433)	0.96	0.305	(0.828, 1.830)	1.23	0.229	(0.532, 1.163)	0.79	0.389	(0.808, 1.370)	1.18	**0.006** ^‡^	(1.164, 2.458)	1.69
	Clinical	–	–		–	–		–	–		–	–		–	–		–	–	
Economy	Average	0.806	(0.634, 1.426)	0.95	0.100	(0.468, 1.065)	0.72	0.722	(0.632, 1.374)	0.93	0.057	(0.470, 1.011)	0.69	0.096	(0.501, 1.058)	0.73	0.881	(0.716, 1.476)	1.03
	Higher	–	–		–	–		–	–		–	–		–	–		–	–	
Abroad Experience	No	0.136	(0.415, 1.127)	0.68	0.126	(0.418, 1.113)	0.68	0.066	(0.389, 1.030)	0.63	0.101	(0.416, 1.082)	0.67	0.079	(0.412, 1.049)	0.66	0.223	(0.480, 1.187)	0.75
	Yes	–	–		–	–		–	–		–	–		–	–		–	–	
Knowing fatal disease	No	0.920	(0.698, 1.384)	0.98	0.192	(0.577, 1.117)	0.8	0.398	(0.626, 1.205)	0.87	0.408	(0.633, 1.204)	0.87	0.314	(0.622, 1.165)	0.85	0.143	(0.586, 1.080)	0.8
	Yes	–	–		–	–		–	–		–	–		–	–		–	–	
Knowing limit daily activity	No	0.658	(0.651, 1.312)	0.92	**0.017** ^†^	(1.078, 2.124)	1.51	0.832	(0.742, 1.450)	0.87	0.194	(0.895, 1.731)	0.24	0.644	(0.672, 1.279)	0.93	0.974	(0.728, 1.360)	0.99
	Yes	–	–		–	–		–	–		–	–		–	–		–	–	
Chi-square		**20.95** ^‡^			**31.75** ^§^			**28.99** ^§^			**24.76** ^‡^			**17.29** ^†^			**17.76** ^†^		

† Statistically significant difference with a p-value < 0.05.

‡ Statistically significant difference with a p-value < 0.01.

§ Statistically significant difference with a p-value < 0.001.

**Table 4.** Ordinal logistic regression analysis from attitudes of Indonesian medical students (Q9–Q11).

Parameters		Q9 GT as future therapy			Q10 GT funding			Q11 GT application in Indonesia		
		p-value	95% CI	OR	p-value	95% CI	OR	p-value	95% CI	OR
Gender	Males	**0.006** ^‡^	(1.139, 2.191)	1.58	0.087	(0.960, 1.833)	1.33	0.012	(1.096, 2.112)	1.52
	Females	–	–		–	–		–	–	
Domicile	Main Islands	0.780	(0.068, 1.310)	0.96	**0.010** ^‡^	(1.103, 2.062)	1.51	0.756	(0.766, 1.443)	1.05
	Other Islands	–	–		–	–		–	–	
Faith	Majority	0.085	(0.514, 1.044)	0.73	0.220	(0.564, 1.141)	0.8	0.816	(0.730, 1.492)	1.04
	Minority	–	–		–	–		–	–	
Studentship status	Pre-clinical	0.269	(0.845, 1.832)	1.24	0.683	(0.739, 1.587)	1.08	0.378	(0.571, 1.238)	0.84
	Clinical	–	–		–	–		–	–	
Economy	Average	0.247	(0.549, 1.167)	0.8	0.199	(0.539, 1.137)	0.78	0.873	(0.664, 1.416)	0.97
	Higher	–	–		–	–		–	–	
Abroad Experience	No	**0.000** ^§^	(0.238, 0.616)	0.38	0.161	(0.449, 1.143)	0.72	0.074	(0.406, 1.043)	0.65
	Yes	–	–		–	–		–	–	
Knowing fatal disease	No	0.638	(0.674, 1.273)	0.93	0.137	(0.574, 1.079)	0.79	0.054	(0.529, 1.005)	0.73
	Yes	–	–		–	–		–	–	
Knowing limit daily activity	No	0.231	(0.592, 1.135)	0.82	0.364	(0.841, 1.603)	1.16	0.538	(0.799, 1.539)	1.11
	Yes	–	–		–	–		–	–	
Chi-quare		**33.09** ^§^			**21.17** ^‡^			**16.37** ^†^		

† Statistically significant difference with a p-value < 0.05.

‡ Statistically significant difference with a p-value < 0.01.

§ Statistically significant difference with a p-value < 0.001.

## Discussion

Good health and well-being are one of the United Nations' Sustainable Development Goals. Even though measurement of well-being remains debatable, the medical world usually focuses on the quality of life including physical, psychological, emotional, social and spiritual dimensions [31]. Many studies have shown that genetic diseases bring physical, financial and psychosocial burdens to the patients, family members and society [32–35]. Several approaches have been proposed and applied to reduce this burden, including genetic testing, health promotion, and the development of new drugs, including gene therapy.

Several gene therapies have been approved as treatments for rare genetic diseases such as Eteplirsen for DMD [19], Zolgensma and nusinersen for SMA [36,37], Luxturna for inherited retinal dystrophy [17], LentiGlobin or Zynteglo for thalassemia [21], CAR-T cell for leukemia and lymphoma [16], and many more in progress [38–40]. Since their launch, debates on their clinical values and price remain ongoing and some studies have endeavored to show the benefit of these approved gene therapies. A computational study showed the benefits of Luxturna in the earlier stages of choroideremia among young adults [41]. Moreover, a study among 26 patients with SMA receiving nusinersen reported an increasing quality of life of the patients [42]. This result differed with a study among 11 patients with SMA in Saudi Arabia receiving nusinersen, for which improved quality of life could not be concluded [43]. Improved quality of life also has been reported among ten patients with thalassemia receiving LentiGlobin [44].

Even though gene therapy development started decades ago, and several gene therapies have been approved during the last 5 years. Our study found that Indonesian medical students had limited knowledge on this technology, as only 37.3% of pre-clinical students and 23.4% of clinical students ever heard about approved gene therapy which did not alter the genomic sequence. This result was lower than their knowledge level on genome editing technology, a part of gene therapy that alters the genome sequence, for which 41.1% of 521 medical students ever heard about this technology [25]. A study among 597 medical and postgraduate students in China showed only 15.54% learned in detail about gene therapy, even though more had heard about this technology [45].

Even though the respondents had limited knowledge about approved gene therapy, almost 80% of respondents supported applying this technology to treat genetic diseases both for fatal and limiting daily activity genetic diseases. Their acceptance of the approved gene therapy was higher than medical doctors' and students' acceptance of genome editing technology which could alter the human genome (60–61%) [25]. Similar to another study, our studies showed declining support for genetic enhancement application (30.57%) which can develop the human appearance and increase ability. This number was comparable with other studies on gene therapy and genome editing from various countries and respondents [46].

Similar to the study on attitudes of Indonesian medical doctors and students concerning genome editing technology, respondents were also concerned about the safety and the price of the approved gene therapy. This finding was similar to that of other studies among 13,201 respondents in China, reporting that 16.4% of clinicians emphasized safety as the most significant concern [26]. Moreover, as the largest Muslim country, 15% of respondents also emphasized that this technology was against religious values and nature. Our results supported the conclusion of a study among 467 US and Canadian respondents revealing less than 20% of respondents agreed that the most concerning matter was being against nature and their religious beliefs. In this study, the respondents' biggest concern was the lack of information regarding this technology [47].

Our models showed only sex might affect almost all attitudes toward approved gene therapy applications for health-related matters. The respondents' domicile also might affect attitudes concerning this technology especially on genetic enhancement and research funding for gene therapy development in Indonesia. While student status might correlate with their agreement on gene therapy for genetic enhancement, the pre-clinical students were more likely to support this purpose. Study abroad experiences also constituted one of the positive predictors in the respondents' belief that gene therapy will be a future therapy.

Most Indonesian medical students remain unaware of new treatments for genetic diseases that might escalate the health and well-being of children with genetic diseases and family members. Several studies have reported increased quality of life after receiving approved gene therapy treatment [41–44], so more efforts are needed to expand the knowledge concerning this technology to the future doctors in Indonesia. However, safety and access, including economic access, must be assured before applying in Indonesia.

As a web-based survey was used in this study, so bias caused by unfamiliarity with the internet and unavailable internet connections should be considered. A study with wider respondents and qualitative studies are needed to explore medical students' attitudes toward approved gene therapy.

Gene therapy is a new breakthrough for the medical world which provides a solution for diseases that no treatment has yet been found. It could improve children with a genetic disease and their family well-being and quality of life. There is a possibility that gene therapy will be used in Indonesia, therefore preparedness for adopting this new treatment, consideration on safety, ethical issues, cost and accessibility should be taken into account. This study provides information about the acceptance of Indonesian medical students on this technology that can be used as a basis for policymaking.

## Conclusion

This study discovered the unfamiliarity of Indonesian medical students about gene therapy. Despite their unfamiliarity, they mostly agree on the application of health-related matter such as treating genetic diseases. Their acceptance was determined by the sex, domicile and studentship status. Since there is a possibility of gene therapy application in Indonesia, increasing medical students’ knowledge of gene therapy is important to minimize the conflict that might occur, especially regarding the safety and ethical issues.

**Summary points**

- Indonesian medical students have little knowledge of gene therapy.
- Majority of Indonesian medical students support the use of gene therapy for fatal diseases and diseases that limit daily activities but it is reduced with purposes to genetic enhancement, such as appearance, intelligence and strength.
- Indonesian medical students are concerned about the safety, price and the effect of gene therapy which is against religious beliefs and nature.
- The acceptance was determined by gender, domicile, study abroad experience and studentship status.
